# Unmasking the Silent Threat: Prevalence of Cardiovascular Risk Factors in MS Patients in Bucharest, Romania

**DOI:** 10.3390/jcm14062001

**Published:** 2025-03-15

**Authors:** Florentina Cristina Pleșa, Elena Andreea Arsene, Andreea Pleșa, Săndica Bucurică, Daniela Anghel, Carmen Adella Sîrbu, Monica Marilena Țânțu, Alexandru Andrei Badea, Octavian Vasiliu, Alice Elena Munteanu

**Affiliations:** 1 Clinical Neurosciences Department, ‘Carol Davila’ University of Medicine and Pharmacy, 050474 Bucharest, Romania; cristina.plesa@umfcd.ro (F.C.P.); carmen.sirbu@umfcd.ro (C.A.S.); 2 Department of Cardiology, University Emergency Hospital Bucharest, 050098 Bucharest, Romania; elenaarsene28@gmail.com; 3 Doctoral School, Faculty of Medicine, “Carol Davila” University of Medicine and Pharmacy, 050474 Bucharest, Romania; 4 Department 5, Internal medicine and Gastroenterology, “Carol Davila” University of Medicine and Pharmacy, 020021 Bucharest, Romania; 5 Department of Internal Medicine, Central Military Emergency University Hospital, 010242 Bucharest, Romania; daniela.anghel@prof.utm.ro; 6 Department of Medico-Surgical and Prophylactic Disciplines, Faculty of Medicine, Titu Maiorescu’ University, 031593 Bucharest, Romania; 7 Academy of Romanian Scientists, 050045 Bucharest, Romania; 8 Department of Medical Assistance and Kinesiology, Faculty of Sciences, Physical Education, and Informatics, University Center of Pitești, 110040 Pitești, Romania; marilena.tantu@upb.ro; 9 National University of Science and Technology Politehnica, 060042 Bucharest, Romania; 10 Department of Cardiology, ‘Dr. Carol Davila’ Central Military Emergency University Hospital, 010825 Bucharest, Romania; badea.alexandru97@gmail.com (A.A.B.); dralicepopescu@yahoo.com (A.E.M.); 11 Department of Psychiatry, “Dr. Carol Davila” University Central Emergency Military Hospital, 010825 Bucharest, Romania; octavvasiliu@yahoo.com; 12 Department of Medical-Surgical and Prophylactical Disciplines, Faculty of Medicine, ‘Titu Maiorescu’ University, 031593 Bucharest, Romania

**Keywords:** multiple sclerosis, cardiovascular risk, smoking, dyslipidemia, glycemia, arterial hypertension, renal function, EDSS

## Abstract

**Background and Objectives:** This study aims to identify cardiovascular risk factors among 91 patients with multiple sclerosis in Bucharest, Romania, using data from medical records and a self-administered questionnaire. **Materials and Methods:** The research focuses on smoking status, body mass index, blood sugar, cholesterol, triglycerides, blood pressure, and renal function. The average age of the patients was 42.90 ± 10.60 years, and 74.73% were female. The average BMI was 23.89 kg/m^2^ ± 4.74 kg/m^2^ (lower than in the general population), with 41.76% of patients having a BMI outside normal limits. The majority were former smokers (35.87%), followed by non-smokers (28.26%), and the fewest were smokers of more than 20 cigarettes/day (7.61%). The average total cholesterol was 198.48 ± 38.56 mg/dL, triglycerides were 114.22 mg/dL ± 76.2281 mg/dL, and blood glucose was 97.54 ± 36.58 mg/dL. While 25.27% of the patients had fasting blood glucose > 126 mg/dL, only five patients were diagnosed with DM. The average blood pressure was 137.5165 ± 19.5047 mmHg, with only 11 patients diagnosed with HTA. **Results:** The study found an average cardiovascular risk of 6.6133% ± 7.1412%, with higher values among males, rural patients, those with secondary-progressive MS, and those with a disease progression >10 years (after age adjustment). **Conclusions:** The study underscores the importance of identifying and combating cardiovascular risk factors in patients with multiple sclerosis.

## 1. Introduction

Multiple sclerosis (MS) represents a significant public health concern, being the primary cause of non-traumatic neurological disability among young adults both in Romania and globally, with an estimated 2.8 million individuals affected [1]. MS is a severe, chronic, and progressive disease that affects the central nervous system (CNS), with predominantly cerebral lesion [2], and the manifestations of the disease have been described since the mid-19th century. The disease involves both motor and sensory manifestations, as well as cognitive impairments that worsen over time. The etiology of inflammatory and degenerative processes remains elusive, attributed to the interaction of environmental and genetic factors affecting the immune system [3].

While previous studies have examined cardiovascular risk factors in patients with multiple sclerosis (MS), our research addresses a specific gap in the existing literature by focusing on the prevalence of these risk factors among MS patients in Romania. This population is underrepresented in current research, and our study provides unique, region-specific insights into the potential geographical and demographic influences on cardiovascular risk within the MS community.

The course of a patient with MS is unpredictable and challenging to anticipate [3]. Both relapses and remissions are difficult to predict [4]. The diagnosis is established by associating clinical features (signs and symptoms) with the presence of lesions on paraclinical investigations, respecting the dissemination in time and space according to the McDonald diagnostic criteria [4,5,6], and by excluding other pathologies.

Cardiovascular diseases (CVDs) account for approximately one-third of global mortality, a significant portion of which is avoidable and occurs prematurely. CVDs, mainly ischemic heart disease (IHD) and stroke, are the foremost contributors to global illness, thereby exacerbating the disabilities experienced by individuals with MS [7]. The distress endured by patients leads to elevated disability-adjusted life years (DALYs), a reduced quality of life, and frequently substantial medical costs. The effects on patients also encompass societal and economic consequences. Absences or diminished efficacy at work result in reduced output, imposing a burden of lost productive time. This problem extends beyond paid employment; declines in productivity also impact unpaid labor, obstructing tasks such as cleaning, childcare, and volunteering, all of which are vital for a functional community [8].

Patients diagnosed with MS have been shown to have a 3.5-fold greater risk of death from any cause and a 1.5-fold higher risk of death from cardiovascular disease [9]. As regards the prevalence of cardiovascular risk factors (hypertension, diabetes mellitus, smoking, dyslipidemia, etc.) in patients with MS compared to the general population, the data in the literature are inconsistent, with no clear consensus [7,8,10].

## 2. Materials and Methods

### 2.1. Type of Study

This study is a descriptive, observational, retrospective, cross-sectional analysis. Its objective is to identify cardiovascular risk factors and assess their prevalence among 91 patients with MS who were hospitalized in the Neurology Department of the Central Military Emergency University Hospital in Bucharest between September 2022 and September 2023.

### 2.2. Study Population

Patients were selected according to the criteria in Table 1.

### 2.3. Data Collection

The data for this study were collected by reviewing patient observation forms from the hospital’s archive, which had been initially gathered through anamnesis, clinical examinations, and paraclinical investigations. From these records, we utilized the following data: age, sex, personal and family medical history, MS phenotype, age of disease onset, body mass index (BMI), smoking status, alcohol consumption, treatment details, glycemia, and lipid profile (including cholesterol, HDL, LDL, and triglycerides).

Additionally, data were collected using a self-administered questionnaire designed to identify potential cardiovascular risk factors. Meticulously developed by our research team, the questionnaire was specifically tailored to gather the detailed information we required. This custom approach was chosen over existing validated questionnaires because it allowed for the collection of extensive and precise data on the patients’ lifestyles, which other tools did not fully cover. The questionnaire was distributed both online and in physical format.

The first question explicitly sought the patient’s consent for the use of their data while ensuring their anonymity. It read: “Do you agree that the data collected through this questionnaire may be used for statistical research and educational purposes to assess the dynamic status of health and associated pathologies?” (Ro: “Sunteți de acord ca datele dumneavoastră colectate prin acest chestionar să fie utilizate în scopuri statistice de cercetare și didactice pentru evaluarea în dinamică a stării de sănătate și a patologiei asociate?”). This ensured that patients were fully informed about the purpose of data collection and could voluntarily provide consent, while maintaining the confidentiality of their information.

The processing of patient data was conducted in accordance with international regulations, legislation, and ethical standards. Consent to access patients’ medical data was obtained from the hospital’s ethics committee (approval number: CR 6344, issued on 1 July 2023).

The study was conducted in accordance with the Declaration of Helsinki and received approval from the Ethics Committee of the Central Military Emergency University Hospital. Informed consent was not required for the retrospective data used in this article, which were obtained from patient observation sheets (including medical history and lab results), as the data were collected retrospectively. These data were used exclusively for research and statistical purposes, adhering to ethical standards for retrospective studies. To estimate cardiovascular (CV) risk over a 10-year period, we utilized the SCORE algorithm, developed by the European Society of Cardiology (ESC). This tool assesses the risk of coronary atherosclerosis and major cardiovascular events, such as heart attacks and strokes [11].

### 2.4. Methods

Once the data were collected, they were systematically divided into several sections: baseline characteristics, lifestyle risk factors, medical history, blood tests, and SCORE algorithm.

**Baseline Characteristics:** This section included variables such as sex, age, disease duration (time from diagnosis to the present), area of residence, disease phenotype, and EDSS score. To simplify reporting and use disability levels as a marker for disease severity, we classified patients with an EDSS score of <2.5 as having a mild disease phenotype, while those with a score of ≥2.5 were categorized as having a more severe phenotype. We selected this cut-off because, on the EDSS scale, it represents the transition from minimal disability, which does not impact the patient’s daily life, to a more severe form that affects their quality of life. This classification allowed us to efficiently assess the relationship between disease severity and cardiovascular risk factors.

**Medical History:** To analyze personal medical history, we used data from both the observation sheets and the self-administered questionnaires completed by the patients. We focused specifically on personal medical conditions relevant to cardiovascular risk, including diagnosed cardiovascular diseases such as hypertension and atherosclerosis (thickening or hardening of the arteries caused by plaque buildup in the artery walls), a history of acute myocardial infarction, history of stroke, recent episodes of angina within the last three months, and the presence of diabetes mellitus. This comprehensive approach allowed us to capture all pertinent aspects of the patients’ medical histories related to cardiovascular risk.

**Lifestyle Risk Factors:** To assess smoking habits, patients were categorized into five groups: non-smokers, ex-smokers, passive smokers (non-smokers regularly exposed to cigarette smoke), and current smokers who smoke either ≥20 or <20 cigarettes per day. This classification allowed for a more detailed analysis of smoking-related risks. Additionally, patients’ psychological status was evaluated based on self-reported stress levels (using our custom questionnaire to capture psychological details without being exhaustive) over the past six months, as well as the frequency of anxiety and depression episodes. Physical activity levels were also self-rated and divided into four categories: sedentary (minimal activity, mostly sitting or watching TV), light activity (slow walking, housework), moderate activity (walking more than 30 min daily, cycling, dancing), and intense activity (regular sports like jogging, cycling, or yoga). This comprehensive approach enabled a thorough evaluation of lifestyle risk factors in the cohort.

**Blood Tests:** We selected only the blood analyses available for all patients and calculated the mean values for each parameter. The analyses included glycemia, cholesterol levels, HDL, LDL, and triglycerides. To ensure consistency and accuracy, we used the biological reference intervals provided by the hospital’s laboratory, where the analyses were conducted.

**SCORE Algorithm:** Given the wide variation in disease duration within the study group, patients were divided into two categories: those with less than 10 years of disease duration and those with more than 10 years. The 10-year cut-off was selected because the mean disease duration in the study group was 9.14 ± 5.86 years. This stratification was essential for accurately assessing the impact of disease duration on cardiovascular risk. While these two groups were not ideal due to discrepancies in phenotype, EDSS, and sex, which should be explored in future studies, we chose this division due to the small sample size. To ensure accurate statistical analysis, patients under 40 years of age were excluded from the SCORE calculation, as their SCORE is 0%.

### 2.5. Statistical Analysis

The collected data were stored and subsequently processed and statistically analyzed using Microsoft Office Excel 2021. Both categorical variables (including binary, nominal, and ordinal data) and quantitative variables (comprising discrete and continuous data) were included in the analysis. We conducted a descriptive analysis to understand the overall distribution and characteristics of the data. For categorical variables, we calculated the frequency distribution to determine the number of occurrences for each category. For quantitative variables, we identified the range, as well as the minimum and maximum values, to capture the spread and extremes of the data.

To summarize the central tendencies within the dataset, we computed the mean (average) for the quantitative variables. In addition, we calculated standard deviations and standard errors to assess the variability and precision of the mean values, providing insights into the consistency and reliability of the data.

To determine the statistical significance of the processed data, we employed the Student’s *t*-test for quantitative variables. This test was used to compare the means between two groups and assess whether the observed differences were statistically significant. A *p*-value was calculated for each comparison, with a value of less than 0.05 considered statistically significant. A statistically significant *p*-value suggests that the observed differences between groups are unlikely to have occurred by chance, thereby supporting the validity of our findings.

These analyses were meticulously performed to ensure a detailed and accurate understanding of the data, thereby strengthening the reliability and validity of the study’s conclusions.

## 3. Results

Of the 108 patients who met the inclusion criteria for the study, 17 of them were eliminated due to exclusion criteria: three for criterion 1, two for criterion 2, eleven for criterion 3, and one for criterion 4. The data for this study were collected by reviewing patient observation forms from the hospital’s archive, which had been initially gathered through anamnesis, clinical examinations, and paraclinical investigations. From these records, we utilized the following data: age, sex, personal and family medical history, MS phenotype, age of disease onset, body mass index (BMI), smoking status, alcohol consumption, treatment details, glycemia, and lipid profile (including cholesterol, HDL, LDL, and triglycerides).

### 3.1. Baseline Characteristics (Table 2)

Among the 91 patients included in the study, 68 (74.73%) were female, reflecting the gender distribution of MS cases in the general population [12]. The mean age of the cohort was 42.90 ± 10.60 years. The largest subgroup of patients was aged 30–45 years, comprising 40 individuals (43.96%), followed by those over 45 years with 39 individuals (42.86%), and the smallest subgroup, consisting of 12 individuals (13.19%), was under 30 years. The mean age at disease onset was 33.86 ± 9.81 years. The mean duration of disease within the study group was 9.14 ± 5.86 years, with a range from 8 months to 28 years.

**Table 2:** Baseline characteristics of study population. * Relapsing–remitting multiple sclerosis. ** Secondary-progressive multiple sclerosis. *** Primary-progressive multiple sclerosis.

Baseline Characteristics	
**Sex**	74.73% female, 25.27% male
**Age**	Mean: 42.90 ± 10.60 years 30–45 years: 43.96% >45 years: 42.86% <30 years: 13.19%
**Age at disease onset**	Mean: 33.86 ± 9.81 years
**Duration of disease**	Mean: 9.14 ± 5.86 years
**Area of residence**	71.43% urban areas, 28.57% rural areas
**MS phenotype**	84.62% RRMS *, 10.99% SPMS **, 4.40% PPMS ***
**Expanded Disability Status Scale**	Mean: 2.60 ± 1.82

Most of the patients, 65 (71.43%), resided in urban areas, while the remaining 26 (28.57%) were from rural areas, yielding an urban-to-rural ratio of 3:1.

Regarding the characteristics of the disease, the relapsing–remitting phenotype of MS was the most prevalent, accounting for 84.62% of cases. This was followed by the secondary-progressive form, which constituted 10.99% of cases, and the primary-progressive phenotype, which was the least common at 4.40%.

We assessed the degree of disability in patients using the Expanded Disability Status Scale (EDSS), with a mean score of 2.60 ± 1.82.

### 3.2. Lifestyle Risk Factors

Most of the patients were former smokers (33, 35.87%), followed by non-smokers (26, 28.26%), and the fewest were smokers of more than 20 cigarettes/day (7, 7.61%).

Regarding alcohol consumption, the results are encouraging: 40% of patients reported never drinking alcohol, while another 40% stated that they consume alcohol monthly or less frequently.

**Figure 1:**
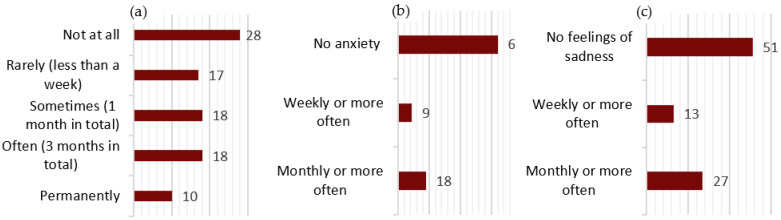
(**a**) Level of stress; (**b**) frequency of anxiety episodes; (**c**) frequency of depression episodes.

About one third of the patients (31.86%) considered themselves to be stressed for more than 3 out of 6 months or even permanently, 34 of the patients (37.36%) said that they were sometimes or rarely stressed, and the rest (30.77%) were not stressed (Figure 1a). Symptoms that suggest panic attacks were experienced by 29.67% of patients, 66.66% of whom reported panic attacks on a weekly basis (Figure 1b). Feelings of sadness, depression, hopelessness, loneliness, or loss of interest in social interaction were present in almost half (43.95%) of the patients in the group, with a third (32.50%) of them reporting their presence weekly or more often (Figure 1c).

Given that body weight is a significant cardiovascular risk factor, we assessed each patient’s body mass index (BMI) and abdominal circumference. The mean BMI was 23.89 ± 4.74 kg/m², while the mean abdominal circumference was 83.51 ± 17.71 cm. More than half of the patients (53, 58.24%) had a BMI within the normal range. However, six patients (6.59%) were classified as underweight, twenty-five (27.47%) as overweight, five (5.49%) as having grade II obesity, and two (2.20%) as having grade III obesity.

The mean BMI of participants was analyzed based on age group and sex. The lowest mean BMI (22.94 ± 4.03 kg/m²) was observed in the 30–45 age group, while similar mean BMIs were recorded for patients under 30 (24.55 ± 3.25 kg/m²) and those over 45 (24.65 ± 5.64 kg/m²), but not with statistical significance. A statistically significant difference (*p* < 0.05) emerged when stratifying by sex, with females having a lower mean BMI compared to males (23.41 ± 4.61 kg/m² vs. 25.31 ± 4.91 kg/m², respectively).

Approximately 26% of participants indicated a moderate level of physical activity, and only 6% fell into the intense activity category. We then averaged the EDSS score for each category. The sedentary patients had the highest mean EDSS score (4.9545), and those with intense physical activity had a mean EDSS score of 1.500, statistically significant (*p* < 0.05). Physical activity level was not correlated with patients’ BMI, with all groups having a mean BMI within normal limits (Table 3). It was evident that the degree of disability served as a restrictive factor for the level of activity, but no correlation between the BMI and the level of physical activity was observed.

### 3.3. Medical History (Figure 2)

For analyzing personal pathological history, we used both data from the observation sheets and data provided by the patients through the questionnaire.

**Figure 2:**
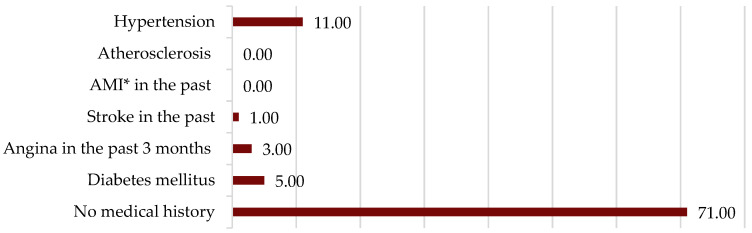
Medical history. * AMI = acute myocardial infarction.

On clinical examination, including a single blood pressure measurement, the mean blood pressure values were 137.52 ± 19.50 mmHg for systolic blood pressure (sBP) and 84.67 ± 22.88 mmHg for diastolic blood pressure (dBP). Although only 11 patients had a formal diagnosis of hypertension, 44 (48.35%) exhibited systolic BP ≥ 140 mmHg or diastolic BP ≥ 90 mmHg, and 30 (32.97%) had borderline high BP (120–139/80–89 mmHg).

**Figure 3:**
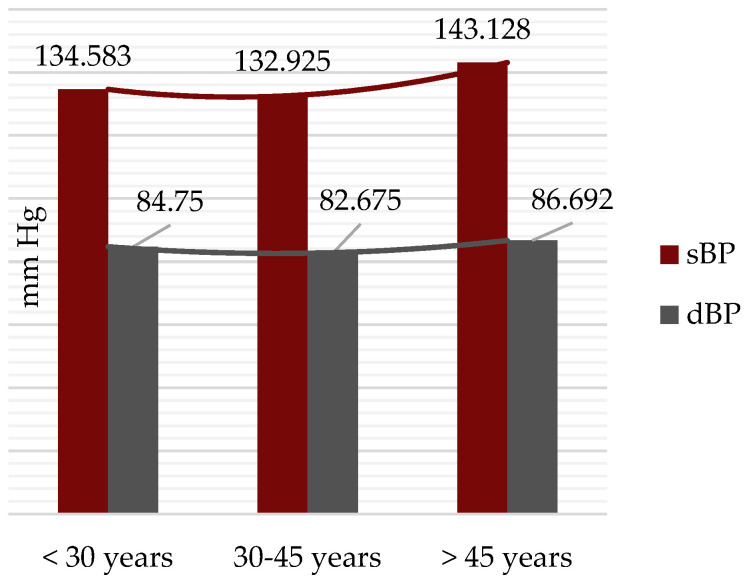
Mean systolic and diastolic blood pressure stratified by age group.

Stratifying by age group, the mean sBP and dBP values were calculated. Patients aged 30–45 years had the lowest values (sBP = 132.83 mmHg, dBP = 82.68 mmHg), whereas those over 45 years had the highest values, with means exceeding the maximum accepted normal values (sBP = 143.13 mmHg, dBP = 86.69 mmHg), but not with statistical significance (Figure 3).

### 3.4. Blood Tests (Table 4)

Although only five patients in the group had a formal diagnosis of diabetes mellitus (DM), an additional five patients exhibited blood glucose levels ≥ 126 mg/dL, suggesting possible undiagnosed DM. The mean total cholesterol value among the patients was 198.48 ± 38.56 mg/dL, which is close to the upper limit of normal. According to reference values, 51 patients (56.04%) had normal cholesterol levels, 27 patients (29.67%) were at moderate cardiovascular risk (200–239 mg/dL), and 13 patients (14.29%) were at high risk (values > 240 mg/dL).

**Table 4:** The mean value of selected medical analyses from patients’ observation sheets.

Blood Test	Mean	Standard Deviation	Biological Reference Intervals
**Glycemia** **(mg/dL)**	97.5948	36.5822	74.00–100.00
**Total Cholesterol** **(mg/dL)**	198.4817	38.5618	<200—normal 200–239—moderate risk >240—high risk
**LDL-cholesterol** **(mg/dL)**	120.8003	36.6784	<100—normal 100–129—low risk 130–159—moderate risk >160—high risk
**HDL-cholesterol** **(mg/dL)**	56.6636	14.9341	10–40 (male)/50 (female) high risk 41/51–90 normal
**ESR (erythrocyte sedimentation rate)** **(mm/1 h)**	13.4407	10.9580	0.00–20.00
**Creatinine** **(mg/dL)**	0.7369	0.1711	0.51–0.95
**Urea** **(mg/dL)**	29.3637	8.5922	17.00–43.00

The mean LDL-cholesterol value was 120.80 ± 36.67 mg/dL. Approximately one-third of the patients had normal values, while those at low risk (100–129 mg/dL) were nearly equal in number to those at moderate risk (130–159 mg/dL). Only seven individuals had values above 160 mg/dL, indicating high risk.

**Figure 4:**
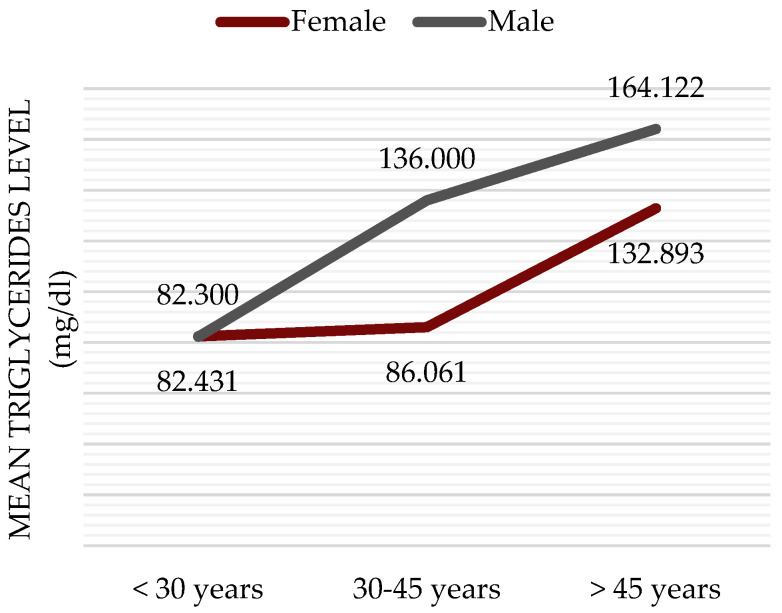
Age- and sex-specific triglyceride curves.

The mean HDL-cholesterol value was 56.66 ± 14.93 mg/dL, with most patients having normal values. According to reference values, twenty-one women (30.88%) had HDL-C values < 50 mg/dL, and three men (13.04%) had HDL-C values < 40 mg/dL, placing them at major cardiovascular risk. Regarding triglyceride levels, an increase in the mean value was observed in direct proportion to the age of the patients. Patients under 30 years of age had the lowest mean value (82.39 ± 21.56 mg/dL), while those over 45 years of age had a mean value of 140.10 ± 95.79 mg/dL, and this was statistically significant (*p* < 0.05). When dividing the group by sex and age, an approximately linear increase in mean triglyceride values was observed in men, whereas a sharp increase in triglycerides occurred among women after the age of 45 years (Figure 4).

We focused exclusively on the erythrocyte sedimentation rate (ESR), as it was the only marker consistently available in the patients’ blood tests. The mean value of ESR was 13.4407 ± 10.9580 mm/s. Notably, patients with the secondary-progressive phenotype of MS exhibited the highest mean value of the erythrocyte sedimentation rate (ESR) at 21.0000 mm/s, in comparison to 12.8065 mm/s for the relapsing–remitting phenotype and 6.7500 mm/s for the primary-progressive phenotype.

Recognizing the myriad factors that contribute to the decline in renal function of MS patients, including age, comorbidities such as hypertension or diabetes, and the adverse effects of treatments, presents a challenge in isolating the impact of each individual factor. We analyzed the serum creatinine and urea, which had values of 0.7369 mg/dL and 34.8428 mg/dL, respectively. No significant difference was observed between the age groups. Patients with systolic blood pressure exceeding 130 mmHg demonstrated higher mean creatinine and urea levels, though the difference was not statistically significant (*p*-value > 0.05).

### 3.5. SCORE Algorithm

The Romanian population was categorized as being at high CV risk. Thus, depending on age, sex, smoking/non-smoking status, non-HDL cholesterol, and systolic blood pressure values, we calculated the SCORE value. The mean value of the cardiovascular risk obtained was 5.9670% ± 6.8774% (MIN = 0%, MAX = 31%). Mean cardiovascular risk in men (6.4782 ± 8.8824%) was higher than in women (4.7941 ± 6.1244%).

A significant difference was observed in the patients’ backgrounds, with individuals from rural areas exhibiting a cardiovascular risk of 7.9629%, compared to a much lower mean risk of 5.1250% among urban residents (*p* = 0.048).

We analyzed the influence of dyslipidemia on the SCORE value. Although the non-HDL-cholesterol value is incorporated into the SCORE algorithm, we aimed to assess the impact of the accumulation of lipid metabolism disorders on cardiovascular risk. Accordingly, we calculated the risk for each patient group: those without dyslipidemia, those with hypercholesterolemia alone, those with hypertriglyceridemia alone, and those with both conditions. Patients with both hypertriglyceridemia and hypercholesterolemia demonstrated the highest cardiovascular risk based on the SCORE value (*p*-value = 0.0855, not statistically significant) (Table 5).

We divided patients again, this time according to EDSS score as we presented at the beginning of this study. We averaged cardiovascular risk for each group of patients. The results showed that patients with a lower degree of disability had a SCORE value approximately half that of those with more severe disability, and the difference was statistically significant (*p* < 0.05).

**Figure 5:**
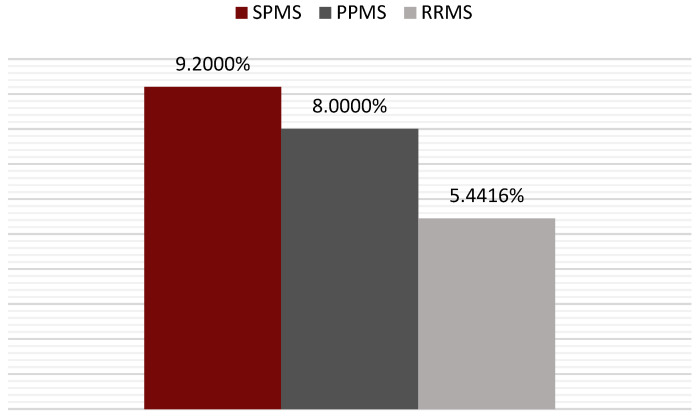
Mean SCORE for each form of multiple sclerosis.

Patients with relapsing–remitting MS had the lowest risk based on the SCORE value, with a mean SCORE value of 5.441%, and those with secondary-progressive MS (SPMS) reached a value of 9.200%, only 1% higher than that of those with primary-progressive MS (PPMS) (*p* >0.05—not statistically significant) (Figure 5).

We evaluated the impact of disease duration on SCORE value. To assure that the current age does not dictate the SCORE value, we tested the influence of current age using a *t*-test, and there was no significant difference in terms of age between the groups that could affect cardiovascular risk (*p* > 0.05). Consequently, we proceeded with the statistical analysis of the estimated cardiovascular risk for the two categories. The results indicated, with statistical significance (*p* < 0.05), that individuals with a disease duration longer than 10 years had a higher cardiovascular risk.

## 4. Limitations

This study has several limitations that should be considered when interpreting the results. First, the sample size was relatively small, consisting of 91 patients. Such a cohort size may limit the statistical power of the study and restrict the generalizability of the findings to a broader population. Additionally, all participants were recruited from a single country—Romania—which may introduce geographical and ethnic biases. The homogeneity of the sample could impact the external validity of the study, as cultural, environmental, and genetic factors specific to this region might influence the prevalence of cardiovascular risk factors among patients with multiple sclerosis (MS). Despite these limitations, the consistency of our results with existing literature suggests that the findings remain reliable and provide valuable insights into the cardiovascular health of MS patients.

Secondly, the retrospective design of the study imposed limitations on the depth and scope of the data collected. Relying on existing medical records meant that we could only utilize the information that was previously documented. This approach may have resulted in incomplete data for certain variables and a lack of standardized measurements across all patients. For instance, detailed lifestyle factors or additional inflammatory markers might not have been consistently recorded, thereby constraining our ability to perform a more comprehensive analysis. The inherent limitations of retrospective data collection could introduce bias and affect the accuracy of the results.

Thirdly, we used the original Systematic Coronary Risk Evaluation (SCORE) model instead of the updated SCORE2, which was introduced in 2021 to account for both cardiovascular mortality and morbidity. The decision to use the original SCORE model was based on two main factors: the availability of relevant variables in our dataset and the limited number of studies employing SCORE2 at the time of our research. Using the original SCORE model enabled us to compare our findings with existing literature more effectively. However, this choice may have resulted in an underestimation of cardiovascular risk in our patient population, as SCORE2 offers a more comprehensive risk assessment. The lack of widespread adoption of SCORE2 in contemporary studies restricted our ability to use it without compromising the comparability of our results.

Looking forward, future research should aim to address these limitations by including larger, more diverse cohorts that encompass multiple geographical regions. A prospective study design would allow for the collection of standardized and comprehensive data, including additional biomarkers like homocysteine and C-reactive protein (CRP), which could provide a more complete picture of systemic inflammation and cardiovascular risk in MS patients. Furthermore, adopting the SCORE2 model in future studies would enhance the accuracy of cardiovascular risk assessment and facilitate comparison with emerging literature.

Overall, while acknowledging these limitations, we believe that our study provides valuable insights into cardiovascular risk factors in patients with multiple sclerosis. Our findings emphasize the importance of cardiovascular risk assessment in this population and highlight the need for increased clinical awareness and the development of preventive strategies.

## 5. Discussion

The significant predominance of females among individuals with MS is a notable aspect of the disease. Although men are generally considered to have a higher cardiovascular risk, certain life stages in women, such as pregnancy and menopause, along with hormone replacement therapies and pregnancy-related pathologies, can temporarily increase the risk of major cardiovascular diseases [13,14]. This suggests that cardiovascular risk in women with MS may be underestimated, particularly due to the specific physiological changes they experience. Therefore, it is essential to consider these factors when analyzing cardiovascular risk in this population, given that the majority of MS patients are female, including in our cohort [15].

The 3:1 ratio between the two areas of residence may be due to a lack of attendance and limited access to health services for people in rural areas. Another factor may be the migration of young people to urban areas with the aging of the rural population, the average age of urban people being 42.33 years compared to 45.20 years for rural areas.

The elevated number of non-smokers and ex-smokers compared to current smokers among the study participants may be attributed to the strong recommendation to cease smoking upon diagnosis of MS. However, these figures are not significantly different from those observed in the general population of Romania according to the data from Eurostat in 2019 [16].

Upon comparison of our findings with those of a study that used the Depression, Anxiety, and Stress Scale-21 (DASS-21) to assess MS patients, the results show similar levels of stress and anxiety. However, our study group exhibited lower levels of depression [17]. A possible explanation for this discrepancy could be the patients’ perception of quality of life, which affects the answers to the questionnaire. According to a Eurostat report published in October 2023, Romania ranks third in Europe for quality of life, with a score of 7.7 [18].

The BMI values observed align with literature data, showing that the average BMI is lower in MS patients compared to the general population of the same age and sex. This difference can be attributed to muscle atrophy and the increased metabolic rate commonly seen in individuals with MS [19,20,21].

Notably, when comparing these findings with data from the literature, the percentage of MS patients diagnosed with hypertension is similar to reported figures [22,23,24], but the percentage of those with elevated blood pressure values is significantly higher, closely mirroring the prevalence of hypertension in the general adult population in Romania [23]. It is important to acknowledge potential limitations related to blood pressure values, such as the influence of white-coat hypertension, which could affect the accuracy of single-point measurements in certain individuals. Referring to the blood pressure analysis for each age group, the slightly concave shape of the mean blood pressure curve (Figure 3) may be attributed to increased awareness of cardiovascular risk among patients aged 30–45 years and the subsequent initiation of antihypertensive treatment within this group.

Notably, there appears to be a higher proportion of MS patients who have not yet been diagnosed with hypertension and diabetes mellitus [24,25,26]. The prevalence of elevated LDL-C appears to be lower among MS patients compared to the general population [27,28]. The literature shows wide variability in the prevalence of low HDL-cholesterol values [28], making it difficult to determine whether the results obtained are consistent with existing data.

Our study showed that patients with both hypertriglyceridemia and hypercholesterolemia had the highest cardiovascular risk. This underscores the importance of targeting multiple lipid abnormalities in managing cardiovascular risk (Table 5).

Treatment options for MS have expanded significantly since the introduction of interferons, with the development of various disease-modifying therapies (DMTs) that target different mechanisms of the disease [29]. These include monoclonal antibodies such as natalizumab and ocrelizumab, oral agents like fingolimod, dimethyl fumarate, and teriflunomide, and newer infusion therapies such as alemtuzumab. These DMTs aim to reduce relapses, slow disability progression, and limit new disease activity visible on MRI [30]. The early initiation of effective disease-modifying therapy, aimed at reducing inflammation in the initial years following onset, can decrease the risk of cerebrovascular events [9]. However, it is unclear to what extent these therapies directly influence cardiovascular risk, as some medications have side effects such as hypertension or hyperglycemia. For example, fingolimod has been associated with bradyarrhythmia and hypertension [31], while siponimod has been linked to an increased risk of bradyarrhythmia [32]. Additionally, dimethyl fumarate can cause flushing and palpitations, and teriflunomide may lead to hypertension [33]. Monoclonal antibodies like alemtuzumab can cause infusion-related reactions, an increased risk of infections, and autoimmune complications. Rare but severe side effects include immune thrombocytopenic purpura [34], hepatitis, cardiovascular complications, and malignancies in some cases (e.g., with cladribine) [35,36]. Consequently, managing the cardiovascular side effects of these DMTs is crucial for maintaining overall cardiovascular health in MS patients. Patients should be closely monitored for cardiovascular changes, and healthcare providers should consider these potential side effects when designing comprehensive treatment plans.

Due to all patients in our study being part of the national multiple sclerosis treatment program with disease-modifying therapies (DMTs), we were unable to analyze the impact of DMTs on the lipid profiles of the patients. The existing literature on this topic is limited. However, one study evaluating real-life data from six different DMTs (interferon-beta, glatiramer acetate, teriflunomide, dimethyl fumarate, fingolimod, and ocrelizumab) concluded that these therapies had no effect on serum lipid levels [28,37]. Research continues to advance, with numerous drugs in development focusing on more targeted and effective approaches, including those that promote remyelination and neuroprotection. DMTs play a crucial role in altering the natural course of MS, improving long-term outcomes, and preserving quality of life.

Patients with MS show a blood triglyceride concentration pattern similar to that of the general population, with a sharp increase observed in females during the postmenopausal period [15], highlighting the need for careful monitoring and assessment.

The elevated ESR levels observed in patients with secondary-progressive MS may suggest the presence of inflammatory “flare-ups” and increased disease activity, as supported by the literature [28,38]. To draw more definitive conclusions, a comprehensive study that includes additional inflammatory markers (such as C-reactive protein and homocysteine), along with MRI imaging to confirm disease activity and assessments to rule out other potential causes of inflammation and elevated ESR (including infections and secondary effects of medication), is warranted.

The percentage of patients with DM in this study was lower than that reported in a similar cohort (35.5%) [25]. When compared to the prevalence of DM type II in Romania, the results are comparable in terms of the percentage of diagnosed diabetics (8.4–11.6%) [26].

Disease progression in secondary-progressive MS (SPMS) and primary-progressive MS (PPMS) is characterized by a steady increase in disability, which may contribute to a higher cardiovascular risk. Chronic inflammation, more persistent in SPMS and PPMS, can exacerbate cardiovascular issues. Patients with progressive phenotypes of MS may also experience more limitations in physical activity, leading to a sedentary lifestyle and increased cardiovascular risk. Furthermore, comorbidities such as hypertension and diabetes are often more prevalent in patients with progressive MS, further elevating their cardiovascular risk. Lastly, the long-term effects of treatments for MS could also contribute to an increased cardiovascular risk in these patients.

The SCORE algorithm was updated in 2021 to the SCORE2 version, which calculates the risk of mortality as well as morbidity and contains four population categories—low, moderate, high, and very high—and replaces the total cholesterol values with non-HDL cholesterol values. In the new algorithm, Romania moves from the category of high-risk countries to that of very high risk, with a value approximately double the estimated risk for each person, and the difference between the risk value for women and men decreases considerably [39]. Because SCORE2 was published in October 2021 and the studies conducted using this new score are very few, even more so if we refer only to those done in Romania, we decided to use the old algorithm in this study, to compare the results with the data from the literature. Nonetheless, the exponential increase in cardiovascular risk for each patient remains a significant concern, underscoring the need for a thorough understanding of its severity.

Approximately six out of one hundred patients with MS are at risk of developing cardiovascular disease in the next 10 years, double compared to the general population in Romania.

The higher cardiovascular risk among urban patients may be due to the majority of young patients under 40 years of age living predominantly in urban areas. The combination of hypertriglyceridemia and hypercholesterolemia elevates the risk of cardiovascular disease more than a single lipid disorder, potentially by exacerbating other cardiovascular risk factors like high blood pressure.

Comparing the results with respect to the characteristics of MS and cardiovascular risk, we can see that they are consistent with those obtained in a study that calculated cardiovascular risk using the Framingham score in patients with MS [40].

Although our study did not utilize modern non-invasive imaging techniques capable of uncovering latent cardiovascular pathologies [41]—such as supra-aortic trunk ultrasound, echocardiography, cardiac magnetic resonance imaging, and coronary computed tomography angiography—we acknowledge their significant importance in the comprehensive assessment of cardiovascular risk. We recognize that incorporating these advanced imaging modalities could enhance the detection of subclinical cardiovascular diseases in patients with multiple sclerosis. Therefore, we encourage future studies to include these techniques, which may contribute to the development of more refined diagnostic guidelines and improve patient outcomes.

The management of patients with MS is complex and requires a multidisciplinary approach throughout the course of the disease. Although a causal relationship between MS and cardiovascular risk is not fully elucidated, periodic screening for risk factors is essential for preventing cardiovascular and cerebrovascular events, considering the increased risk with aging and possibly with the duration of disease-modifying treatments.

Thus, this study aims to identify and analyze the prevalence of cardiovascular risk factors among patients with multiple sclerosis (MS) and to explore the relationship between cardiovascular risk and the progression of the disease. Recognizing and addressing the impact of these factors on the course of MS offers significant benefits for both the healthcare system and the patients. It allows for more personalized treatment and care. A multidisciplinary approach is essential to address all aspects of treatment—disease modification, symptom management, and recovery—ultimately contributing to the improvement of patients’ quality of life.

## 6. Conclusions

Patients with multiple sclerosis have a higher cardiovascular risk compared to the general population [10]. The prevalence of type II diabetes mellitus among these patients is similar to that of the general population in Romania [26]. The pattern of blood triglyceride concentration evolution in these patients also mirrors that of the general population. Patients with both hypertriglyceridemia and hypercholesterolemia have demonstrated the highest cardiovascular risk based on the SCORE value. Additionally, patients with MS and a higher degree of disability face an increased cardiovascular risk. Those with secondary-progressive MS also exhibit higher cardiovascular risk. A disease duration longer than 10 years correlates with an elevated cardiovascular risk. The relationship between cardiovascular risk and the progression of MS is complex and necessitates extensive research. However, the primary focus of this study is to emphasize the importance of early identification and mitigation of cardiovascular risk factors, alongside advocating for a multidisciplinary therapeutic approach in managing patients with MS.

Table 6 provides a comprehensive summary of the key data, offering enhanced understanding and a clearer visualization of the cardiovascular risk factors analyzed within our study population.

## Figures and Tables

**Table 1:** Inclusion and exclusion criteria for defining study population. * The National Health Program for MS is a government program in Romania that has been designed to ensure access to diagnosis and treatment for patients with multiple sclerosis.

**Inclusion criteria**
Patients diagnosed with multiple sclerosis
Patients who have attained the age of 18 years at the time of enrolment in the study
Patients under treatment through the National Health Program for MS * between September 2022 and September 2023
Patients whose paraclinical investigations are in the hospital record
**Exclusion criteria**
Patients who had serious concomitant diseases (cancer, hepatitis, etc.) or treatments (e.g., chemotherapy) that could interfere with cardiovascular risk (3)
Patients who refused to answer the questionnaire (2)
Women aged ≥ 60 years and men aged ≥ 55 years (11)
Patients with clinically isolated syndrome (CIS) (1)

**Table 3:** Average age, EDSS, and BMI for different groups according to their level of activity.

Level of Physical Activity	Average Age (years)	Average EDSS Score	Average BMI (kg/m²)
**Sedentary (minimal activity, mostly sitting/watching TV)**	45.23	4.9545	21.5350
**Light Activity (slow walking, household chores)**	46.22	2.6300	24.9327
**Moderate Activity (walking more than 30 min daily, cycling, dancing)**	37.75	1.5625	23.1498
**Intense Activity (regular sports: jogging, cycling, yoga, etc.)**	33.89	1.5000	22.6430
***p*-value**	0.0080	0.0009	0.1298

**Table 5:** Mean SCORE according to the association of hypercholesterolemia and hypertriglyceridemia among patients.

Mean SCORE (%)		
	Cholesterol < 200 mg/dL	Cholesterol ≥ 200 mg/dL
Triglycerides < 150 mg/dL	5.4523	5.62068
Triglycerides ≥ 150 mg/dL	4.1111	10.3636

**Table 6:** Characteristics of sample and cardiovascular risk factors.

Risk Factor	Sample Characteristics
**Smoking Status**	Non-smokers: 28.26%, Ex-smokers: 35.87%, Smokers < 20/day: 28.26%, Smokers ≥ 20/day: 7.61%
**Alcohol Consumption**	Never drink: 40%, Consume monthly or less: 40%, Uncertain: 20%
**Stress Levels**	Stressed > 3 months or permanently: 31.86%, Sometimes/Rarely stressed: 37.36%, Not stressed: 30.77%
**Panic Attacks/Depression/Sadness Symptoms**	29.67% of patients had panic attacks (66.66% weekly) 43.95% of patients report symptoms of depression or sadness (32.50% weekly or more)
**BMI**	Mean BMI: 23.89 ± 4.74 kg/m²; 58.24% normal, 6.59% underweight, 27.47% overweight, 7.69% obese
**Physical Activity Level**	Sedentary: 13%, Light: 55%, Moderate: 26%, Intense: 6%
**Blood Pressure (Mean)**	Systolic BP: 137.52 ± 19.50 mmHg, Diastolic BP: 84.67 ± 22.88 mmHg
**Cholesterol (Total)**	Mean: 198.48 ± 38.56 mg/dL; 56% normal, 30% moderate risk, 14% high risk
**LDL-Cholesterol**	Mean: 120.80 ± 36.67 mg/dL; 33% normal, 33% low risk, 34% moderate risk
**HDL-Cholesterol**	Mean: 56.66 ± 14.93 mg/dL; 30% women and 13% men had low HDL (major risk)
**Triglycerides**	Increased with age; patients > 45 years had the highest levels
**Diabetes Mellitus**	Five patients had a formal diagnosis of diabetes mellitus (DM) Five patients showed blood glucose ≥ 126 mg/dL, indicating possible undiagnosed DM
**Inflammatory Markers (ESR)**	Mean ESR: 13.44 mm/h; higher in secondary-progressive MS patients (21.00 mm/h)

## Data Availability

The data supporting the findings of this study are not publicly available due to privacy. Requests for access to the data should be directed to the corresponding author.
